# Physicochemical, Antioxidant and Antimicrobial Properties of Electrospun Poly(ε-caprolactone) Films Containing a Solid Dispersion of Sage (*Salvia officinalis* L.) Extract

**DOI:** 10.3390/nano9020270

**Published:** 2019-02-15

**Authors:** Ana Salević, Cristina Prieto, Luis Cabedo, Viktor Nedović, Jose Maria Lagaron

**Affiliations:** 1 Department of Food Technology and Biochemistry, Faculty of Agriculture, University of Belgrade, Nemanjina 6, 11080 Zemun, Belgrade, Serbia; ana.salevic@agrif.bg.ac.rs (A.S.); vnedovic@agrif.bg.ac.rs (V.N.); 2 Novel Materials and Nanotechnology Group, Institute of Agrochemistry and Food Technology (IATA), Spanish Council for Scientific Research (CSIC), Calle Catedrático Agustín Escardino Benlloch 7, 46980 Paterna, Valencia, Spain; cprieto@iata.csic.es; 3 Bioinicia R&D Department, Bioinicia S.L., Calle Algepser 65, 46980 Paterna, Spain; 4 Polymers and Advanced Materials Group (PIMA), Jaume I University (UJI), Avenida de Vicent Sos Baynat s/n, 12071 Castellón, Spain; lcabedo@uji.es

**Keywords:** poly(ε-caprolactone), sage, electrospinning, nanofibers, active packaging, antioxidant activity, antimicrobial activity

## Abstract

In this study, novel active films made of poly(ε-caprolactone) (PCL) containing a solid dispersion of sage extract (SE) were developed by means of the electrospinning technique and subsequent annealing treatment. Initially, the antioxidant and antimicrobial potential of SE was confirmed. Thereafter, the effect of SE incorporation at different loading contents (5%, 10%, and 20%) on the physicochemical and functional properties of the films was evaluated. The films were characterized in terms of morphology, transparency, water contact angle, thermal stability, tensile properties, water vapor, and aroma barrier performances, as well as antioxidant and antimicrobial activities. Thin, hydrophobic films with good contact transparency were produced by annealing of the ultrathin electrospun fibers. Interestingly, the effect of SE addition on tensile properties and thermal stability of the films was negligible. In general, the water vapor and aroma permeability of the PCL-based films increased by adding SE to the polymer. Nevertheless, a strong 2,2-diphenyl-1-picrylhydrazyl (DPPH**^·^**) free radical scavenging ability, and a strong activity against foodborne pathogens *Staphylococcus aureus* and *Escherichia coli* were achieved by SE incorporation into PCL matrix. Overall, the obtained results suggest great potential of the here-developed PCL-based films containing SE in active food packaging applications with the role of preventing oxidation processes and microbial growth.

## 1. Introduction

Oxidation processes and microbial growth are common causes of food deterioration which result in color changes, texture modifications, development of off-flavor, and loss of nutritional value and quality of foodstuffs [1,2]. In this regard, spices and herbs have been traditionally added to food, not just as flavoring and healing agents, but also as preservatives [3]. Nowadays, many spices and herbs are recognized as sources of bioactive compounds which are able to stabilize free radicals and prevent oxidation processes and/or act as bacteriostatic or bactericidal agents [2,3]. In addition, there is a growing interest from consumers and the food industry for the use of natural active compounds in food preservation due to their synergy, potency, and presumed low side effects when compared to synthetic additives [4]. However, a major drawback for direct food application of extracted active compounds represents their susceptibility to adverse external factors, chemical instability, and interactions with food constituents [5]. Another limiting factor is a rapid actuation and diffusion of active compounds within the bulk of food [6,7]. A promising approach to overcome these problems is the incorporation of active compounds in a polymeric matrix by encapsulation, to provide stability, keep functionality, and increase effectiveness during time, creating a physical barrier between actives and their environment [8].

Many efforts done to prevent food deterioration and achieve higher effectiveness of active compounds have triggered innovations in food packaging. This current trend is reflected in the development of active food packaging with improved functionality. In this context, the passive role of traditional packaging in protecting and marketing of a food product has evolved into a novel function as a carrier of active compounds [9,10]. This novel concept is based on the incorporation of various active agents into a packaging material with the aim to maintain or enhance quality and safety, extend shelf life of a packaged product, and to reduce the packaging related environmental pollution [10,11]. Nowadays, the use of natural active compounds as functional ingredients in active food packaging is highlighted.

Sage (*Salvia officinalis* L.) is a herb widely used in cookery due to its seasoning and flavoring properties as well as in traditional medicine to treat dyspepsia, excessive sweating, age-related cognitive disorders, and throat and skin inflammations [12]. Moreover, sage is one of the herbs with a great potential for use as a functional ingredient for the development of active food packaging due to its well-known antioxidant [13,14], antibacterial [3], and antifungal [14] effects. These beneficial activities are positively related to phenolic compounds [3,14], such as phenolic diterpenoids (carnosic acid, carnosol, rosmanol), phenolic acids (caffeic acid, rosmarinic acid, ferulic acid) and flavonoids (luteolin derivatives, apigenin derivatives), among others [13,14,15]. Essential oils and extracts of *Salvia officinalis* are generally recognized as safe according to the U.S. Food and Drug Administration [16].

While formulating active food packaging, an environmental issue related to an increasing quantity of disposed plastic packaging with unknown biodegradation time should be taken into account. This problem has set a strong challenge towards replacement of non-biodegradable polymers by biodegradable ones, especially for single-use plastic items [17]. In this sense, poly(ε-caprolactone) (PCL), a commercially available and biodegradable aliphatic polyester, could represent an alternative. It is a hydrophobic, semi-crystalline polymer with a low melting point and is miscible with many other polymers [18,19]. The rheological and viscoelastic properties allow an easy processability of PCL [18]. This material is very interesting for packaging applications [19], as well as being used as a carrier of active compounds and development of active materials. For instance, Martínez-Abad et al. studied the potential of PCL for preparation of antibacterial solvent casted films containing cinnamaldehyde and allyl isothiocyanate [20]. Also, PCL electrospun fibers loaded with nettle extract and embedded to whey protein isolate were successfully applied as a bioactive coating to inhibit bacterial growth and extend quality of fresh fish fillets [21].

The design of active packaging materials represents a very dynamic field and a real challenge [10]. In this regard, the electrospinning technique supposes an innovative nanofabrication approach for the development of active food packaging coating and interlayer materials. This approach employs a high-voltage electric field imposed on a polymer solution to create ultrathin mats composed of polymeric fibers with diameters in micro, submicro, and nano range [22,23,24]. Because of a high trapping efficiency and no need for high processing temperatures, the electrospinning technique is very suitable for entrapping active compounds, such as antioxidants [24] and antimicrobials [25] within a fibrous polymer matrix [22]. Fiber-based systems have gained a lot of attention as a way to improve active functionality and achieve an optimal effect during the food storage [6]. Also, characteristics such as cost-effectiveness, continuous fabricating capability, and a facile operating process make the electrospinning technique an excellent candidate for the development of active packaging materials [22].

There are some studies on the incorporation of sage extracts into whey protein isolate [26] and chitosan matrix [2] by means of solvent casting technique. The study on the whey protein isolate-based film has proven that there is great potential of sage extract to be used as the active constituent of food packaging for antioxidant protection of meat products. Nevertheless, there was no information on film properties [26]. On the other hand, the chitosan-based film loaded with sage extract has been evaluated with respect to its physical properties, regardless of its active functionality [2]. Despite those research works, according to our knowledge there is no commercial application of sage extract in commercial food packaging. When compared to casted films, electrospun films would provide more desirable properties, such as homogeneous dispersion of film constituents and better solvent resistance [27]. However, to the best of our knowledge, the use of the electrospinning technique to develop a PCL-sage extract system has not been carried out so far.

The main aim of this study was to develop and characterize active PCL-based films loaded with sage extract. Formulations containing different sage extract loadings were prepared, employing the electrospinning technique followed by annealing treatment. A comprehensive investigation was performed to assess the effect of the sage extract addition on morphology, transparency, hydrophobicity, thermal stability, tensile properties, water vapor and aroma barrier performance, and, most importantly, on antioxidant and antimicrobial activities of the films.

## 2. Materials and Methods

### 2.1. Materials

Sage (*Salvia officinalis* L.) was supplied by the Institute for Medicinal Plants Research “Dr. Josif Pančić” (Belgrade, Serbia). Poly(ε-caprolactone) (Mn=80,000), 2,2-diphenyl-1-picrylhydrazyl (DPPH), (±)-6-hydroxy-2,5,7,8-tetramethylchroman-2-carboxylic acid (Trolox) and D-limonene were obtained from Sigma Aldrich (St. Louis, MO, USA). Ethanol, chloroform, butanol, and methanol were purchased from Panreac Quimica SLU (Barcelona, Spain). Müller Hinton Broth and Agar (MHB and MHA) were provided by Merck KGaA (Darmstadt, Germany). Resazurin sodium salt was procured from MP Biomedicals, LLC (Solon, OH, USA). Phosphate buffered saline (PBS) was purchased from Amresco, LLC (Cleveland, OH, USA). All chemicals were used as received without any further purification.

### 2.2. Preparation of Sage Extract

Sage extract (SE) was prepared by maceration. An aqueous solution of ethanol (50% *v*/*v*) was used as extraction medium. Drug to solvent ratio was 1:20. The extraction process was carried out on an orbital shaker (Stuart SSL1, Staffordshire, UK) with continuous agitation fixed at 200 rpm for 90 min at room temperature. The obtained extract was filtered through a medical gauze and the solvent was evaporated by casting in a fume hood (Flores Valles, Madrid, Spain). Sage extract powder was stored under refrigeration conditions prior to use.

### 2.3. Characterization of Sage Extract

#### 2.3.1. Antioxidant Activity

The antioxidant capacity of SE was estimated using the DPPH^·^ free radical scavenging assay [28], with some modifications. SE (10 mg) was dissolved in 1 mL of methanol. Thereafter, 100 µL of the appropriate diluted solution was mixed with 1.9 mL of a DPPH methanolic solution (0.094 mM). A control sample was prepared by adding 100 µL of methanol to 1.9 mL of the DPPH solution. The free radical^·^ scavenging ability was determined by measuring the absorbance at 517 nm after incubation in dark at room temperature for 30 min. An UV/Vis spectrophotometer (model 4000, Dinko instruments, Barcelona, Spain) was used. Percentage of DPPH^·^ inhibition was calculated following Equation (1):

(1) $$I\left(\%\right)=\frac{\mathrm{Ac}-\mathrm{As}}{\mathrm{Ac}}\times 100$$

where Ac is the absorbance of the control and As is the absorbance of the sample. Trolox (0–1000 µM) was used as a standard to prepare calibration curve. The antioxidant activity was determined from the calibration curve and expressed as mM Trolox equivalents (TE)/g SE.

#### 2.3.2. Antimicrobial Activity

The antibacterial properties of SE and subsequently of the films were ascertained in triplicate against *Staphylococcus aureus* (ATCC 6538P) and *Escherichia coli* (ATCC 25922). The bacterial strains were obtained from the Spanish Type Culture Collection (CECT, Valencia, Spain) and stored in PBS with 10% MHB and 10% glycerol at −80 °C. To prepare fresh inoculum, a loopful of the bacteria was cultivated in MHB at optimal growth conditions overnight and an aliquot was again transferred to MHB and grown at 37 °C to the mid-exponential phase of growth. Suspensions containing approximately 5 × 10^5^ CFU/mL were used for antimicrobial activity assays. Previously to each assay, the samples were sterilized by UV radiation for 30 min in a Biostar cabinet (Telstar S.A., Madrid, Spain).

The antibacterial potential of SE was evaluated by the broth microdilution method [29]. 90 µL of the bacterial suspension was added into a 96-well microtiter plate (Thermo Fischer Scientific, Roskilde, Denmark) containing 10 µL of two-fold serially diluted extract (concentration range from 0.31 to 80 mg/mL). Wells containing only MHB and wells with the bacterial suspension in MHB were used as positive and negative controls, respectively. The plates were incubated at 37 °C for 24 h. Afterward, 10 µL of resazurin, a metabolic indicator, was added into each well and incubated at 37 °C for 3 h. Minimum inhibitory concentration (MIC) was considered as the lowest extract concentration that inhibited bacterial growth according to a resazurin color. The contents from the wells containing dilutions designated as MIC were sub-cultured on MHA. Minimum bactericidal concentration (MBC) was established as the lowest extract concentration for which no bacterial growth was observed after incubation at 37 °C for 24 h.

### 2.4. Preparation of Poly(ε-caprolactone) Based Films

#### 2.4.1. Preparation of Solutions for Electrospinning

Plain PCL solution (10% *w*/*w*, designated as PCL) was prepared by dissolving PCL pellets in a solvent comprising chloroform and butanol (chloroform:butanol = 3:1, *v*/*v*) under magnetic stirring at room temperature. Three PCL-based active systems containing different contents of the sage extract (5%, 10%, and 20% *w*/*w* with respect to the polymer content) were formulated. These formulations were designated as PCL-SE5, PCL-SE10, and PCL-SE20, respectively. The solutions were prepared by dissolving the required amount of the sage extract in the chloroform-butanol mixture and stirring overnight. Subsequently, the solutions were centrifuged at 10,000 rpm for 10 min. Supernatants were collected, filtered through 0.22 µm polytetrafluoroethylene (PTFE) filters, and filled up to the initial weight. Afterwards, polymer was added to the solutions and stirred until it was completely dissolved.

#### 2.4.2. Characterization of the Solutions

The viscosity was measured using a rotational viscometer Visco Basic Plus L (Fungilab S.A., Sane Feliu de Llobregat, Spain). The surface tension was determined applying the Wilhemy plate method an Easy Dyne K20 tensiometer (Krüss GmbH, Hamburg, Germany). The conductivity was measured using a conductivity meter (HI98192 portable meter HANNA Instruments, Gothenburg, Sweden). The measurements were made in triplicate at room temperature.

#### 2.4.3. Electro-Hydrodynamic Processing

The preparation of the fibrous mats was carried out using a high throughput Fluidnatek LE-500 pilot line electrospinning equipment with temperature and relative humidity control in the lab mode with a single needle injector (Bioinicia S.L., Valencia, Spain). The solutions were drawn in a 20 mL plastic syringe that was placed on a syringe pump and connected by PTFE tube to a stainless steel needle (20 Gauge). A positive electrode of a high voltage power supply was coupled to the needle. The solutions were electrospun at the constant flow rate of 3 mL/h and the voltage of 19 kV for 2 h. The fibers were homogenously deposited onto an aluminum foil sheet placed on a metallic collector using a scanning injector. The distance between the needle tip and the collector was 15 cm. The process was performed at 25 °C and 30% RH.

Subsequently, an annealing step was applied in order to obtain ultrathin, transparent, and continuous films by fibers coalescence. This process was carried out using a hydraulic press (4122 model, Carver Inc., Wabash, IN, USA) at temperatures 55 °C for 25 s without pressure. The annealing, carried out below the polymer melting point at 60 °C, was optimized for PCL electrospun fibers in a previous work [25]. The films were stored before physical characterization in a desiccator containing dried silica gel at 0% RH and 25 °C.

### 2.5. Characterization of The PCL Based Films

#### 2.5.1. Film Thickness

Before further analysis, the film thickness was measured at five random points of each sample by a digital micrometer with an accuracy of 0.001 mm (S00014, Mitutoyo Corporation, Kawasaki, Japan).

#### 2.5.2. Morphology

Scanning electron microscopy (SEM) analysis of the electrospun fibers and the cryofractured annealed films was performed employing a Hitachi S-4800 microscope (Tokyo, Japan). The films were cryofractured using liquid nitrogen. The samples were attached to beveled holders using a double-sided adhesive tape, coated with a gold-palladium mixture under vacuum, and examined using an accelerating voltage of 5 kV. The average fiber diameter was determined using ImageJ program (National Institutes of Health, Bethesda, MD, USA).

#### 2.5.3. Transparency

The light transmission spectrum was determined by measuring the light absorption in the wavelength range of 200–800 nm using the spectrophotometer (model UV/Vis 4000, Dinko instruments, Barcelona, Spain). Triplicates of the film specimens of 20 mm × 50 mm were fixed to a test cell perpendicularly to the light beam. The transparency (T) was calculated following the Equation (2):

(2) $$T\left({\mathrm{mm}}^{-1}\right)=\frac{A_{600}}{L}$$

where A_600_ is the absorbance measured at 600 nm and L is the film thickness (mm).

#### 2.5.4. Water Contact Angle

The contact angle of water on the film surface was measured using an optical tensiometer (Theta Lite, Staffordshire, UK). Five droplets (5 µL) of ultrapure water were placed on different positions of three species (20 mm × 50 mm) of each sample and the mean values of the contact angle were calculated.

#### 2.5.5. Thermal Analysis

Thermogravimetric analysis (TGA) was performed by a TG-STDA thermobalance (TGA/STDA851e/LF/1600 model, Mettler-Toledo, LLC, Columbus, OH, USA). The samples (~13 mg) were heated from 25 to 700 °C at a heating rate of 5 °C/min under a dynamic nitrogen atmosphere (flow rate 50 mL/min). The measurements were done in five replicates.

#### 2.5.6. Mechanical Properties

Tensile tests were carried out, employing an universal testing machine (AGS-X 500 N model, Shimadzu, Kyoto, Japan) according to the ASTM Standard D638 [30]. The samples were conditioned at 25 °C and 50% RH for 24 h and cut in dumbbell shaped specimens (5 mm × 25 mm). The tests were performed at a crosshead rate of 10 mm/min on five replicates of each film. The stress–strain curves were prepared on the basis of the force–distance data and used to determine elastic modulus, tensile strength, elongation at break, and toughness.

#### 2.5.7. Water Vapor Permeability (WVP)

WVP was measured gravimetrically in triplicate, following the ASTM E96-95 method [31]. Payne permeability cups of 3.5 cm diameter (Elcometer Sprl, Hermelle-sous-Argenteau, Belgium) were filled with 5 mL of distilled water. The films were sealed with silicon rings in the cups and exposed to 100% RH on one side without direct contact with water. The cups were placed in a desiccator at 0% RH and 25 °C and weighted periodically (±0.0001 g) until the steady state was reached. The cups containing aluminum foil with water and PCL-based films without water in the cups were used as control samples to estimate losses of water through the sealing and of volatile compounds, respectively. Water vapor permeation rate (WVPR) was determined from the permeation slopes obtained from the regression analysis of weight loss data versus time. The weight loss was compensated by the very marginal losses through the sealing and volatiles. WVPR was multiplied by the film thickness to determine WVP.

#### 2.5.8. D-Limonene Permeability (LP)

LP was determined in triplicate, as previously described for WVP. The Payne permeability cups containing 5 mL of D-limonene and the sealed films were stored at 40% RH and 25 °C. Limonene permeation rate (LPR) was determined from the permeation slopes. LPR was multiplied by the film thickness to determine LP.

#### 2.5.9. Antioxidant Activity

Dynamics of DPPH^·^ free radical inhibition by the films was monitored. 1.9 mL of the DPPH^·^ solution was added into a vial containing 1 mg of film sample. Control sample contained 1.9 mL of the DPPH^·^ solution without film. Inhibition rate was determined by measuring the absorbance at 517 nm after incubation of the vials at 100 rpm in dark at room temperature for 0.5, 6, 12, and 24 h. The results were obtained following the Equation (1) and expressed as percentage of DPPH^·^ inhibition. All measurements were performed in triplicate.

#### 2.5.10. Antimicrobial Activity

The antibacterial activity of the films was assessed using a modified Japanese Industrial Standard (JIS) Z2801 [32]. The bacterial suspension was spread between the film samples (dimensions of 2 cm × 2 cm). The inoculated film samples were incubated at 95% RH and 25 °C for 24 h. Thereafter, the bacteria were recovered with PBS and plated onto MHA. The number of viable cells was determined after incubation at 37 °C for 24 h. The antibacterial activity of the films was defined by surface reduction of log_10_ CFU of the test culture during incubation. Surface reduction (R) was calculated according to the Equation (3):

(3) $$R=\left[\log\left(\frac{B}{A}\right)-\log\left(\frac{C}{A}\right)\right]=\log\left(\frac{B}{C}\right)$$

where A is the average of the viable bacterial counts on the control sample immediately after inoculation, B is the average of the viable bacterial counts on the control sample after 24 h and C is the average of the viable bacterial counts on the test sample after 24 h. According to the value of the surface reduction, the antibacterial activity of the films was evaluated with the following assessment: nonsignificant (R < 0.5), slight (0.5 ≤ R < 1), significant (1 ≤ R < 3), and strong (R ≥ 3).

#### 2.5.11. Statistical Analysis

The results were expressed as mean ± standard deviation. The data were subjected to one-way analysis of variance (ANOVA) using Statgraphics Centurion XV software (StatPoint, Inc., Warrenton, VA, USA). Tukey’s HSD test, at 95% confidence level, was performed to determine the influence of SE addition on the properties of the PCL-based films.

## 3. Results and Discussion

### 3.1. Sage Extract Characterization

#### 3.1.1. Antioxidant Activity

The antioxidant potential of SE was evaluated in terms of DPPH**^·^** free radicals scavenging ability. SE exhibited the free radical inhibition of 50.56% at concentration of 0.5 mg SE/mL, which corresponds to an antioxidant activity of 1.07 mmol TE/g SE. The here-obtained extract presented similar free radical inhibition as some herb extracts well-known for a strong antioxidant activity, such as *Camellia sinensis* L. and *Rosmarinus officinalis* L., which showed 49.47% and 55.32% scavenging effects, respectively, at a concentration of 0.5 mg/mL [33]. Potent antioxidant activity of sage extracts was reported in many studies in which phenolic compounds, i.e., abietane diterpenoids (carnosol and carnosic acid) and caffeic acid derivates (rosmarinic acid, chlorogenic acid, caffeic acid) were marked as the most effective constituents with the free radical scavenging ability [15,34,35]. The antioxidant activity of phenolic compounds is due to hydroxyl groups positioned along the aromatic phenolic ring, which act as hydrogen or electron donors enabling termination of free radical chain reactions, as well as from the aromatic ring which is able to stabilize and delocalize the unpaired electron [36,37]. In accordance with the presented results, SE may be used as a functional constituent of novel packaging materials to prevent oxidation reactions.

#### 3.1.2. Antimicrobial Activity

Antimicrobial potential of SE was scrutinized in terms of MIC and MBC values against food-borne pathogens: Gram-positive *S. aureus* and G-negative *E. coli*. The obtained results have shown higher sensitiveness of *S. aureus* to SE with MIC and MBC values of 0.31 and 20 mg/mL, respectively. On the other hand, higher concentrations of SE presented the inhibitory (2.50 mg/mL) and biocidal (40 mg/mL) effects against *E. coli*. According to the literature, the antibacterial activity of SE is highly correlated with phenolic compounds [3]. The mechanism of biocidal action of plant compounds is based on degradation of cell wall, damage to cytoplasmic membrane and membrane proteins, leakage of content out of the cell, and coagulation of the cytoplasm [38]. The noticed higher susceptibility of *S. aureus* as a Gram-positive bacteria is in agreement with the previous study and related to the above-mentioned mechanism of biocidal action. In particular, this behavior originated from differences between cell wall structure of Gram-positive and Gram-negative bacteria. Namely, Gram-negative bacteria possess a more complex cell wall and an outer membrane which acts as a barrier to the penetration of the antimicrobial compounds providing higher resistance [3]. Generally, the obtained results suggest that SE has a potential to be used as a functional ingredient for development of materials with not just antioxidant, but also antibacterial activity.

### 3.2. Solution Characterization

Electrospinning behavior is greatly influenced by the solution properties, typically viscosity, surface tension, and conductivity [6]. Plain PCL solution, as a control sample, and the PCL-based solutions containing SE at three different contents (5%, 10%, and 20% SE) were prepared. As it can be seen in Table 1, the solution parameters are affected by SE addition. A greater reduction in viscosity was observed when the SE content rose. This reduction can be related to the presence of low molecular weight compounds in SE and possible effects on the configuration of polymer chains in the solutions. Furthermore, the addition of a higher amount of SE (10% and 20% SE) significantly increased conductivity. This effect can be attributed to changes in viscosity and mobility of charged species [39]. Regarding the surface tension values, a significant surfactant effect was noticed only for the highest content of SE (20%).

Physical properties of the solutions were suitable for producing continuous fibers under stable electrospinning process without dripping of the solutions or formation of beaded areas (processing parameters are described in Section 2.4.3). Ultrathin films were made by subsequent exposure of the mats to the annealing post-processing treatment.

### 3.3. Film Characterization

#### 3.3.1. Morphology

**Figure 1:**
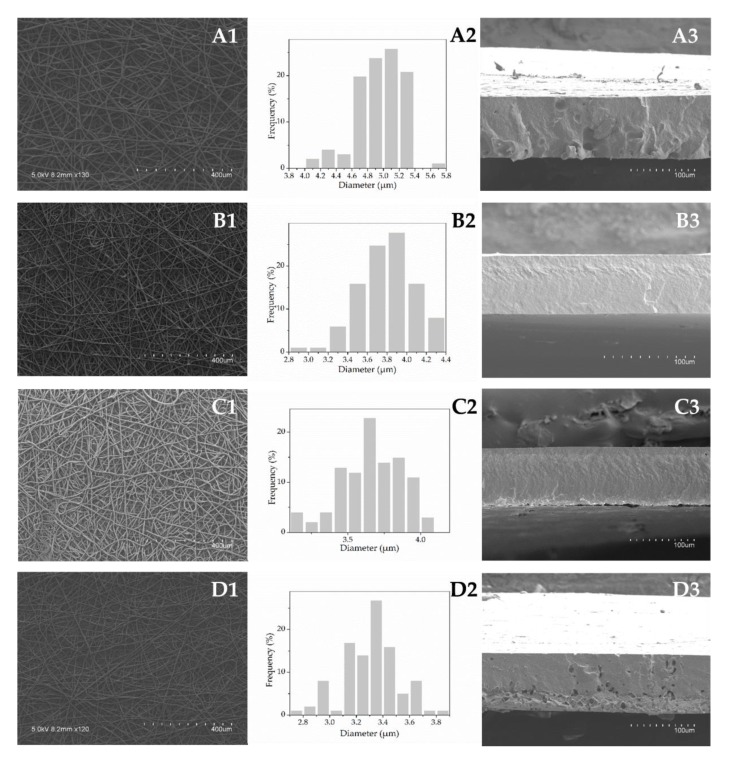
Scanning electron microscopy (SEM) micrographs of the electrospun fibers (A1-D1, vertically), diameter distribution histograms (A2–D2, vertically) and cross-sections of the annealed films (A3–D3, vertically) of the plain poly(ε-caprolactone) PCL (A1–3) and the formulations containing 5% (B1–3), 10% (C1–3), and 20% (D1–3) of SE.

SEM micrographs of the electrospun fibers before the annealing treatment and their corresponding diameter distribution histograms are shown in Figure 1 (A1-D1, A2-D2, vertically). The electrospun mats presented a fibrous, bead-free morphology. The fibers were regular, smooth, and without aggregates, suggesting that a homogeneous solid dispersion of SE is achieved in the fibers (Figure 1, A1-D1, vertically). As shown in histograms (Figure 1, A2-D2, vertically), SE addition affected the fibers’ diameter. It was observed that the increase in SE content led to reduced mean values of fiber diameter (Table 1). This effect is related to the changes in the solution properties induced by SE addition. Likewise, higher conductivity and lower viscosity and surface tension altered the polymer chain entanglements, generating an increase in the stretching forces in the jet which resulted in the decreased electrospun fiber diameters [6,40]. Similarly, decreased diameter of PCL fibers was reported when other active compounds, such as black pepper oleoresins [25], carvacrol [41], or cefazolin [42] were incorporated within the polymeric matrix.

Figure 1 (A3–D3, vertically) presents the cross-sections of the cryo-fractured annealed films. As can be observed, the electrospun fibrous mats were packed into continuous films due to fibers’ coalescence during the annealing treatment. Furthermore, different structures were revealed depending on the SE content. The plain PCL film exhibited a somewhat rougher appearance as compared to the samples containing SE at levels 5% and 10%, which appeared somewhat more compact, smooth, and homogeneous. Higher roughness for the PCL film may be due to the higher fiber diameter. On the other hand, the incorporation of 20% SE led to a more porous morphology. This may be due to the generation of volatiles arising from the higher SE concentration during the annealing process.

Film thickness was found to be lower for the samples with the higher SE content due to most likely lower fiber diameter as a result of a more efficient fiber packing after the annealing treatment (see Table 1).

#### 3.3.2. Optical Properties

**Figure 2:**
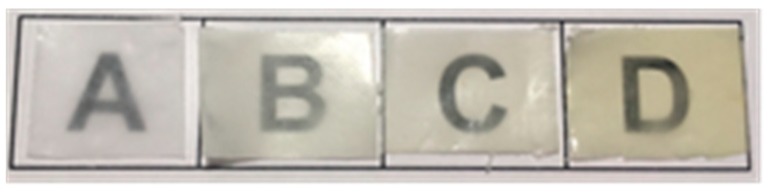
Contact transparency image of the plain poly(ε-caprolactone) PCL film (A) and the PCL-based films containing: 5% (B), 10% (C), and 20% (D) SE.

The developed films were highly transparent, as shown in Figure 2. Film transparency is a desirable property for packaging applications, since the packaging should enable visual assessment of its content. The active films exhibited a yellowish color, which was more intense with increasing the active ingredient content. However, the SE addition did not significantly (*p* > 0.05) alter the transparency, as compared to the control. In particular, the plain PCL film presented a transparency value of 12.96 mm^−1^, while the transparency of the films containing 5%, 10%, and 20% SE was 13.31, 14.24, and 16.16 mm^−1^, respectively. The slight decrease in transparency of the film loaded with the highest extract content (20%) is related to the more heterogeneous morphology and light scattering [43].

Light, especially in the UV range, triggers photo-oxidation processes, which leads to rapid quality loss or deterioration of packaged food products [44]. Evaluation of the UV light transmission capacity implied that, in general, the developed films very effectively blocking UV light. At 300 nm the plain PCL film presented a good light barrier, with a transmission value 1.84%. The incorporation of SE within the PCL matrix significantly helped to decrease penetration of the UV light to very low levels. The light transmission rate at 300 nm was 1.07%, 1.00%, and 0.63% for the films containing 5%, 10%, and 20% SE, respectively. This barrier property makes the studied systems suitable for protection of the products susceptible to photo-oxidation.

#### 3.3.3. Water Contact Angle

Contact angle between a drop of water and the film surface is an indicator of the surface hydrophilicity (θ < 65°) and hydrophobicity (θ > 65°) [45]. The here developed systems present hydrophobic surfaces with poor water affinity (θ > 65°). The plain PCL film presented a contact angle of 76.6° which is in line with the literature (θ ~ 74°) [25]. Interestingly, the film hydrophobicity was increased by increasing the extract content. In particular, the incorporation of 5%, 10% and 20% of SE resulted in the contact angle values of 73.6°, 92.3°, and 100.8°, respectively. This behavior has been ascribed before to changes in surface topology, since an increase in surface roughness and heterogeneity leads to higher values of contact angle [46,47]. Thus, air could be trapped within these micro or submicron size interfiber valleys making air pockets which lead to an increase in water contact angle [48,49]. It is expected that the thinner the fiber and the higher the heterogeneity along the fiber due to increasing SE content, the higher could be the contact angle, as observed. The trend towards greater hydrophobicity of materials containing natural extracts was also reported for starch films with incorporated yerba mate extract, which was explained by observed roughness when the extract was incorporated [50]. The observed water resistance is a highly desirable property for potential food packaging applications.

#### 3.3.4. Thermogravimetric Analysis

**Figure 3:**
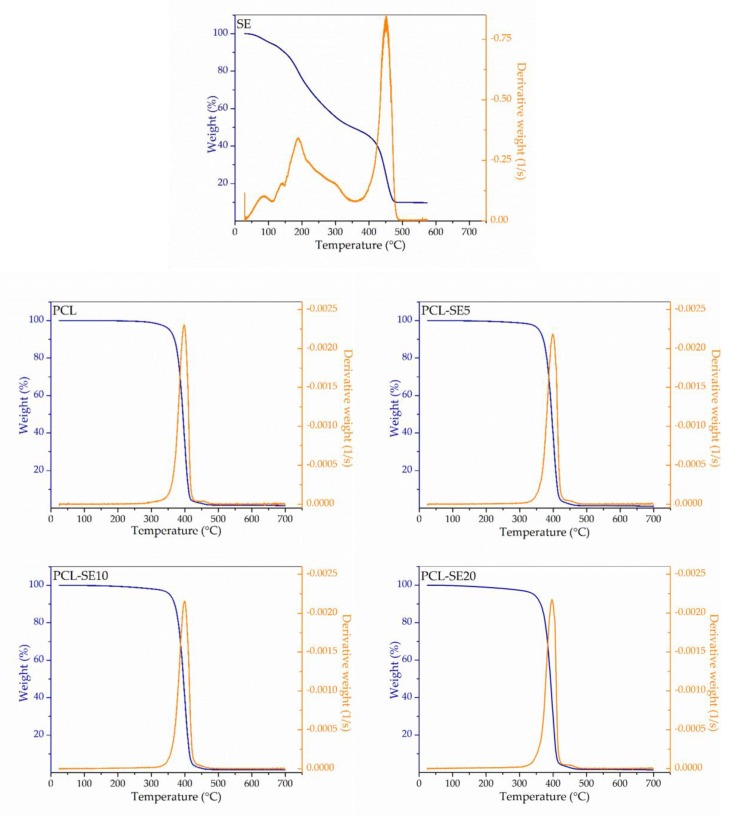
Thermogravimetric curves of the free sage extract (SE) and the poly(ε-caprolactone) (PCL)-based films.

Thermal stability of the free SE and the prepared films was determined by thermogravimetric analysis (TGA). TGA curves of the mass loss as a function of temperature (blue lines) and the first derivative analysis (orange lines) are presented in Figure 3. Degradation of the free SE occurred in several phases, starting from around 106 °C at 5% of weight loss, due to most likely moisture and volatiles evaporation. The maximum degradation rate with a mass loss of about 76% was reached at 452 °C, while residual mass at 600 °C was at about 9%. As it can be seen from the Figure 3, thermal degradation properties of the plain PCL film are not altered by the SE incorporation. The PCL-based films exhibited similar thermal degradation patterns, regardless of the SE content. Generally, the thermal decomposition process of the here-obtained films took place within the range between 350 and 480 °C, largely coinciding with the main degradation of the SE. This thermal degradation range corresponds to the one reported for PCL nanofibrous mat [51]. The maximum degradation rate with a mass loss of about 60% was observed at 398, 399, 399, and 396 °C for the plain PCL film and the films containing 5%, 10%, and 20% SE, respectively. It can be concluded from TGA results that the incorporation of SE into the PCL matrix did not detrimentally affect the thermal stability of the composites when compared to the control sample (without SE).

#### 3.3.5. Mechanical Properties

The tensile properties (elastic modulus, tensile strength, elongation at break, and toughness) of the developed systems are presented in Table 2. The incorporation of SE induced a slight, not statistically significant (*p* > 0.05) decrease in elastic modulus, and an increase in tensile strength, elongation at break, and toughness compared to the PCL film. The observations suggest that the SE, if anything, acts as a very slight plasticizer to PCL. This tensile behavior is in line with the afore-observed thermal behavior. The negligible effect of SE on the mechanical properties of the films indicates that there are not significant interactions between the polymer matrix and the SE compounds [52]. Similarly, mechanical properties of a PCL–gelatin–PCL multilayer system were not significantly affected by addition of black pepper oleoresins into the PCL layers [25]. As compared to a commercial packaging material, the here developed films were more resistant to fracture, but less ductile than LDPE. As reported, the pure LDPE film with a thickness of ca. 44 µm presents a tensile strength and an elongation at break values around 5.88 MPa and 112.39%, respectively [53].

#### 3.3.6. Water Vapor and D-Limonene Permeability

**Figure 4:**
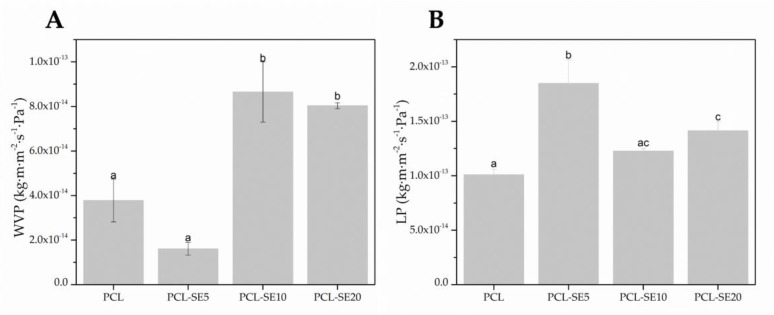
Water vapor (WVP) and D-limonene permeability (LP) of the poly(ε-caprolactone) (PCL)- based films. Different letters within the same column indicate significant differences among samples (*p* < 0.05).

The barrier properties of the materials are relevant for their application, but also to understand the relationship between composition, structure, processing, and properties. The barrier performance of the PCL-based films in terms of water vapor and D-limonene permeability is gathered in Figure 4.

From Figure 4, it can be observed that the plain PCL film presents a higher (*p* < 0.05) water vapor barrier as compared to its counterparts loaded with high extract content (10% and 20%) (Figure 4A). However, the sample with 5% SE loading shows a higher water barrier than neat PCL. This particular sample was seen to have a smoother, less porous morphology than pure PCL. Thus, the reason for the overall changes in permeability to water may be related to sample porosity, but also to SE content. SE may have higher affinity for water than PCL, but at low contents it results in finer fibers that pack better, and at the higher content it produces some porosity due to volatiles leaving the sample during the annealing process. When compared to cellophane as a commercial material widely used in the packaging industry, the plain PCL film and the film containing 5% of SE presented lower water vapor permeability, while the values of the films loaded with 10% and 20% SE were in the same order of magnitude as the value reported for cellophane (6.90 × 10^−14^ kg·m·m^−2^ s^−1^ Pa^−1^) [54].

D-limonene is commonly used as a standard compound to assess the aroma barrier of packaging materials. The SE incorporation was seen to increase the permeability of limonene through the PCL film for all samples (Figure 4B). D-limonene, an apolar permeant, is known to strongly plasticize PCL [49,55]. Therefore, the higher permeability seen for the samples with SE suggests that the comparatively low molar mass of SE facilitates even more the diffusion of the permeant through the materials. The LP of the developed PCL-based films are within the same order of magnitude as the LP of widely used neat poly(ethylene terephthalate) (PET) film produced by compression molding (1.17 × 10^−13^ kg·m·m^−2^ s^−1^ Pa^−1^) [56].

#### 3.3.7. Antioxidant Activity

**Figure 5:**
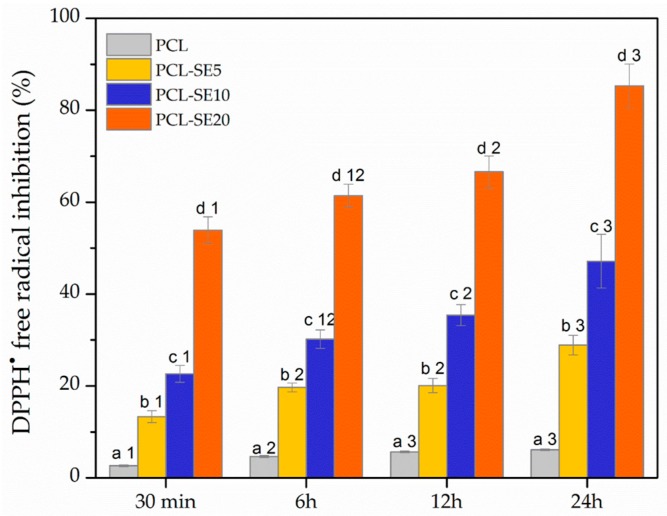
Antioxidant activity, expressed as DPPH**^·^** free radicals scavenging ability of the PCL-based films. Different letters indicate significant difference among different films at certain incubation time (*p* < 0.05). Different numbers indicate significant difference of the same sample at different incubation time (*p* < 0.05).

Figure 5 shows the antioxidant activity of the PCL-based films during 24 h of contact with DPPH**^·^** free radicals solution. According to the results, the unloaded PCL film presented a certain level of antioxidant activity (6.12%) after 24 h. However, the antioxidant activity of the plain PCL film was very weak and might be attributed to a DPPH**^·^** absorption by the film during the contact [51]. The antioxidant activity of the films was achieved when SE was incorporated into the PCL matrix, as expected according to the free radical neutralizing effect of the unloaded SE. Namely, the increase in the loaded SE content led to a significantly stronger (*p* < 0.05) free radical scavenging activity of the films (Figure 5). Also, it may be noted that the DPPH**^·^** free radicals scavenging ability of the studied systems was more pronounced with an increase in the contact time. This behavior can be explained by release of active compounds from the films into the free radicals solution which allows rapid inhibition or quenching of DPPH**^·^** radicals [57]. In particular, the antioxidant activity after 0.5 h of the contact between the free radicals solution and the films loaded with 5%, 10%, and 20% SE was increased from 13.32%, 22.64%, and 53.88%, respectively to 28.89%, 47.17%, and 85.27%, respectively after 24 h. This is in the agreement with a study on chitosan films containing caraway essential oil and beeswax [58] which also reported an increase in antioxidant activity of the films during incubation time. The obtained results point out the efficiency of the PCL-based films containing SE in free radicals neutralization, suggesting their potential role in prevention of oxidation processes in food products.

#### 3.3.8. Antimicrobial Activity

The antibacterial efficiency of the PCL-based films against *S. aureus* and *E. coli* is presented in Table 3. The plain PCL film did not exhibit any antibacterial activity (R < 0.5) as expected. Inhibition effects on the bacterial growth were successfully induced by the incorporation of SE into the PCL matrix. Interestingly, complete biocidal effect on *S. aureus* (no viable counts) was detected after 24 h of exposure to any of the films containing SE. On the other hand, the results indicated a higher resistance of *E. coli* towards the films. This behavior is in concordance with the results obtained for the unloaded extract and attributed to the more complex cell wall of *E. coli* as a Gram negative bacteria compared to the cell wall of *S. aureus* as a Gram positive bacteria. Thus, the inhibition effect on *E. coli* was dependent on the SE content in the films. In particular, *E. coli* was slightly inhibited (0.5 ≤ R < 1) when in contact with the films containing 5% and 10% SE, while strong inhibition (R > 3) was achieved when in contact with the film loaded with 20% SE. Therefore, the strong antibacterial surface against foodborne pathogens was obtained by incorporating 20% SE into the PCL matrix, which reveals its remarkable potential for antimicrobial food packaging applications.

## 4. Conclusions

In the present study, SE solid dispersions with proven antioxidant and antimicrobial activities were successfully incorporated at different loading contents (5%, 10%, and 20%) within the PCL matrix by the electrospinning technique with the aim to develop antioxidant and antimicrobial packaging materials. The electrospun fibrous mats were post-processed by means of the annealing treatment to produce continuous, transparent, active films. The obtained results gave an insight into the effect of SE addition on the physicochemical and functional properties of the PCL-based film. Thus, the properties of the PCL-based solutions and morphology of the formulated films were affected by the SE content. Namely, thinner electrospun fibers were produced when higher SE contents were loaded. Generally, the here obtained systems presented a good contact transparency. UV-light barrier and water resistance, i.e., hydrophobicity, were generally enhanced when SE was loaded within the PCL matrix. The presence of SE had a negligible effect on thermal stability and tensile parameters of the films. On the other hand, SE led to a general decrease in barrier properties to water and D-limonene. Finally, the SE incorporation triggered remarkable DPPH**^·^** free radical scavenging ability and antimicrobial action against *Staphylococcus aureus* and *Escherichia coli*. As the SE content increased, the films presented more effective antiradical ability and activity against *Escherichia coli*. The evaluated characteristics of the PCL-based films containing SE suggest that the here-developed systems are potential candidates as active materials in food packaging applications, which would delay oxidation processes and prevent microbiological contamination.

## Figures and Tables

**Table 1:** Properties of the poly(ε-caprolactone)-based solutions, diameter of the electrospun fibers and thickness of the annealed films.

Formulation	Viscosity (cP)	Conductivity (µS/cm)	Surface Tension (mN/m)	Fiber Diameter (µm)	Film Thickness (mm)
PCL	1908.1 ± 41.2 ^a^	0.02 ± 0.00 ^a^	27.2 ± 0.2 ^a^	4.95 ± 0.29	0.09 ± 0.01 ^a^
PCL-SE5	1695.6 ± 36.6 ^b^	0.02 ± 0.00 ^a^	27.4 ± 0.1 ^a^	3.80 ± 0.28	0.10 ± 0.01 ^a^
PCL-SE10	1579.2 ± 33.7 ^b,c^	0.10 ± 0.00 ^b^	27.5 ± 0.1 ^a^	3.65 ± 0.21	0.08 ± 0.01 ^b^
PCL-SE20	1565.9 ± 35.6 ^c^	0.09 ± 0.00 ^b^	24.7 ± 0.1 ^b^	3.31 ± 0.21	0.08 ± 0.01 ^b^

Data are expressed as mean ± standard deviation. Different letters within the same column indicate significant differences among samples (*p* < 0.05).

**Table 2:** Mechanical properties in terms of elastic modulus (E), tensile strength (σ_b_), elongation at break (ε_b_) and toughness (T) of the poly(ε-caprolactone) (PLC)-based films.

Formulation	E (MPa)	σ_b_ (MPa)	ε_b_ (%)	T (mJ/m^3^)
PCL	420.77 ± 61.45 ^a^	24.32 ± 3.79 ^a^	4.60 ± 0.34 ^a^	1.41 ± 0.19 ^a^
PCL-SE5	425.40 ± 40.85 ^a^	26.11 ± 2.76 ^a^	4.90 ± 0.48 ^a^	1.51 ± 0.09 ^a^
PCL-SE10	410.78 ± 24.70 ^a^	31.98 ± 4.42 ^a^	5.44 ± 0.78 ^a^	1.62 ± 0.02 ^a^
PCL-SE20	359.25 ± 63.43 ^a^	26.43 ± 5.19 ^a^	5.28 ± 0.57 ^a^	1.52 ± 0.15 ^a^

Data are expressed as mean ± standard deviation. Different letters within the same column indicate significant differences among samples (*p* < 0.05).

**Table 3:** Antimicrobial activity against *S. aureus* and *E. coli* of the PCL-based films.

Formulation	*S. aureus*		*E. coli*	
	Bacterial Counts log CFU/mL	Surface Reduction R	Bacterial Counts log CFU/mL	Surface Reduction R
Control	6.10 ± 0.09	-	7.09 ± 0.12	-
PCL	5.77 ± 0.15	0.33	6.89 ± 0.09	0.20
PCL-SE5	no viable counts	biocidal effect	6.36 ± 0.13	0.73
PCL-SE10	no viable counts	biocidal effect	6.18 ± 0.16	0.91
PCL-SE20	no viable counts	biocidal effect	2.40 ± 0.52	4.69

CFU—Colony Forming Units; R—Surface Reduction.
